# Long non-coding RNA LINC00485 acts as a microRNA-195 sponge to regulate the chemotherapy sensitivity of lung adenocarcinoma cells to cisplatin by regulating CHEK1

**DOI:** 10.1186/s12935-019-0934-7

**Published:** 2019-09-12

**Authors:** Wei Zuo, Wei Zhang, Fei Xu, Jing Zhou, Wei Bai

**Affiliations:** Department of Respiration, The First Affiliated Hospital of Nanchang University, No. 17, Yongwaizheng Street, Donghu District, Nanchang, 330006 Jiangxi People’s Republic of China

**Keywords:** Long intervening non-coding RNA LINC00485, MicroRNA-195, CHEK1, Lung adenocarcinoma, Competitive endogenous RNA, Cisplatin, Chemotherapy sensitivity

## Abstract

**Background:**

Long non-coding RNAs (lncRNAs) are a family of non-protein-coding RNAs, which have the ability to influence the chemo-resistance of lung adenocarcinoma (LAC). In this study, we explored the mechanism by which LINC00485 competitively binds to microRNA-195 (miR-195) in the regulation of the chemotherapy sensitivity in LAC by regulating checkpoint kinase 1 (CHEK1).

**Methods:**

Microarray analysis was used to screen out LAC related genes, and interaction between CHEK1 and miR-195, as well as that between miR-195 and LINC00485, was further confirmed by RNA-pull down and RIP. LINC00485 expression in LAC cells (A549 and H1299) was determined. The cells were then introduced with miR-195, anta-miR-195, LINC00485 or si-LINC00485 to identify the role of miR-195 and LINC00485 in LAC through evaluating the expression of CHEK1, CHEK1, Bax, Bcl-2, VEGF and HIF-1α in LAC cells by either RT-qPCR or Western blot analysis. After being treated with different concentration of cisplatin, cell proliferation, colony formation and apoptosis were assessed.

**Results:**

LINC00485 acted as a competitive endogenous RNA against miR-195, and miR-195 directly targeted CHEK1. The expression of LINC00485 was higher in LAC cells. The down-regulation of LINC00485 or the up-regulation of miR-195 decreased the expression of CHEK1, Bcl-2, VEGF and HIF-1α, while also increasing the expression of Bax. Moreover, the over-expression of miR-195, or the silencing of LINC00485 enhanced the sensitivity of LAC cells to cisplatin, thereby promoting the apoptosis of LAC cells while suppressing the proliferation.

**Conclusion:**

LINC00485 competitively binds to miR-195 to elevate CHEK1 expression in LAC cells, suggesting that LINC00485 is a novel direction for therapeutic strategies of LAC.

## Background

Lung cancer, known as one of the most common occurring cancers worldwide, has been shown to be so deadly that it causes many millions of deaths per year [1, 2]. When discussing lung cancer, there are three main kinds of lung cancers that are prevalent to patients worldwide. These three types of lung cancer are; lung adenocarcinoma (LAC), squamous-cell carcinoma and small-cell carcinoma, among which LAC has been found to be the most common type of lung cancers. It has been found that LAC accounts for 40% of all lung cancers, with the morbidity rate increasing in the west and Asia [3]. Because it is the most frequent subtype of non-small cell lung cancer (NSCLC), LAC causes more than 500,000 deaths each year worldwide, carrying with it a poor survival rate of about 15% within five years of the initial diagnosis [4]. In terms of treatment options, computed tomography (CT) screening has been extensively used in order to diagnose early-staged LAC. Furthermore, it is very typical for complete resection to be accompanied by follow-up, with surveillance monitoring usually being adopted thereafter. Nevertheless, majority of patients still suffer from the reoccurring LAC [5]. Moreover, cisplatin has been shown to be essential when treating LAC, with its sensitivity to cisplatin being directly associated to the prognosis [6]. In previous studies, by inhibiting contactin-1, the sensitivity levels of LAC increase, thereby affecting the chemotherapeutic efficacy related to cisplatin based regimens among patients suffering from LAC [7].

Checkpoint kinase 1 (CHEK1) is implicated in regulating and identifying DNA damage [8]. CHEK1 has been shown to be highly expressed in lung cancer cells, and importantly, miR-195 has been demonstrated to inhibit the progression and development of NSCLC by targeting CHEK1 [9]. MicroRNAs (miRNAs) and long noncoding RNAs (lncRNAs) have been shown to have the ability to regulate the ability of regulating each other’s biological progression, particularly, the sponge-like function of lncRNAs, which can lead to complex miRNA interactome [10]. The low expression of miR-195 has an effect on patients suffering from LAC [11]. Furthermore, the noncoding genome elements are involved in normal physiology and human diseases [12]. Among other potential tumor suppressors, differentially expressed lncRNAs have been reported to be among them, when discussing LAC [13]. Moreover, miR-326 has been shown to regulate chemoresistance in LAC cells by binding to specificity protein 1 [14]. Furthermore, miR-195 has been shown to be correlated to chemotherapy sensitivity to cisplatin in gastric cancer cells [15]. Based on the aforementioned findings, we proposed a hypothesis that the LINC00485/miR-195/CHEK1 may be implicated in LAC. And we explored the mechanisms LINC00485 regulating CHEK1 through competitively binding to miR-195 in cisplatin sensitivity of LAC.

## Materials and methods

### Microarray analysis

The profiles of LAC related genes and miRNAs were from the Cancer Genome Atlas (TCGA) database (http://cancergenome.nih.gov/), and analyzed via the R language software. The differential gene analysis used edgeR package in R [16]. False positive discovery (FDR) correction was applied on *p* value with package multi-test. FDR < 0.05 and |log2 (fold change)| > 2 were considered as the screening criteria to select differentially expressed genes (DEGs) and differentially expressed miRNAs.

### Study subjects

The normal human lung epithelial cell line Beas-2B, along with the LAC cell lines A549, H1299, GLC-82 and 95D, were all purchased from Shanghai Cell Bank, Chinese Academy of Sciences (Shanghai, China) and cultured in Roswell Park Memorial Institute (RPMI) 1640 medium containing 10% fetal bovine serum (FBS) at 37 °C with 5% CO_2_. The culture medium was changed every 2–3 days according to cell growth. When cell confluence reached 80%–90%, cells were passaged. The two cells with the highest expression of LINC00485 were screened out by reverse transcription quantitative polymerase chain reaction (RT-qPCR) for the subsequent experiments.

### Cell treatment

The sequences of LINC00485 and miR-195 were retrieved from Genbank. The following plasmids were all constructed by Shanghai Sangon Biotech Company (Shanghai, China), and used to transfect LAC cells; the empty plasmid, LINC00485 plasmid, LINC00485 negative control (NC) plasmid, si-LINC00485 plasmid, miR-195 NC plasmid, miR-195 plasmid, anta-miR-195 NC plasmid and anta-miR-195 plasmid. CHEK1 vectors were purchased from Abcam Inc. (Cambridge, MA, USA).

The day before transfection, the cells were seeded into a 6-well plate. When the density reached 30% to 50%, the transfection was conducted according to the instructions of the lipofectamine 2000 kit. Afterwards, 100 pmol plasmid (final concentration: 50 nM) was diluted with 250 μL serum-free medium (Opti-minimal essential medium [MEM], 51985042, Gibco, Gaitherburg, MD, USA) and mixed slightly and incubated for 5 min, with 5 μL lipofectamine 2000 being diluted with another 250 μL of serum-free medium and mixed gently and incubated for 5 min. Following the incubation period, the plasmid (100 pmol) and the transfection regent (5 μL) were diluted with 250 μL Opti-MEM and incubated for 5 min. The two solutions were mixed, incubated for 20 min, and added to the cells. The two solutions were then mixed together and added to culture wells after 20 min of incubation. Cells were then cultured for 6–8 h, with the medium being changed and continuing to be cultured for 24–48 h.

### RNA fluorescent in situ hybridization (FISH)

The subcellular localization of LINC00485 in LAC cells was identified by FISH according to the instructions of Ribo™ lncRNA FISH Probe Mix (Red) (RiboBio Company, Guangzhou, China). The cover glass was placed in a 24-well plate, and the cells were seeded at a density of 6 × 10^4^ cells/well. The cover glass was fixed with 1 mL 4% polyformaldehyde. Following treatment with protease K (2 μg/mL), glycine, and acetylation reagents, 250 μL of pre-hybridization solution was added to the cells for 1 h of incubation at 42 °C. The pre-hybridization solution was removed, and the cells were incubated with 250 μL of hybridization solution, which contained 300 ng/mL, and was probed at 42 °C overnight. Cells were then added with phosphate-buffered saline/Tween (PBST), and diluted with 4′,6-diamidino-2-phenylindole (DAPI) (1:800) in order to stain the nucleus. Following the staining period, cells were then seeded into a 24-well plate for a staining period which lasted 5 min. Cells were then sealed with anti-fluorescence quencher, observed and photographed under a fluorescence microscope (Olympus, Tokyo, Japan) with 5 different fields.

### Dual luciferase reporter gene assay

In order to predict the target genes of miR-195, a bioinformatics prediction website was used. The 3′-untranslated region (3′ UTR) sequence of CHEK1 and LINC00485 was obtained via PCR amplification. The target segments were digested via the Xho I and Not I restriction endonuclease, and cloned into the pmirGLO vector (3577193, Promega, Madison, WI, USA) on the downstream of the luciferase reporter gene. The vectors were labeled as pCHEK1-wide-type (Wt) and pLINC00485-Wt, with the plasmids being purified following the sequencing period. The sequence of LINC00485-Wt was 5′-TCCACTTTTTCACATTGCTGTTT-3′ and that of CHEK1-Wt was 5′-AATATAGTGCTGCTATGTTGACA-3′. After that, pCHEK1-mutant-type (Mut) and pLINC00485-Mut were constructed. The sequence of LINC00485-Mut was 5′-TCGGCTTAATCACACGATGATGT-3′ and the sequence of CHEK1-Mut was 5′-AATATAGTGCAATGTTGTTGACG-3. The plasmids were co-transfected with miR-195 mimic or miR-195 NC into LAC cells, respectively. The cells were lysed for 24 h following transfection, and centrifuged at 25,764×*g* for 1 min in order to collect the supernatant. The Dual-Luciferase^®^ Reporter Assay System (E1910, Promega, Madison, Wisconsin, USA) was applied in order to measure the luciferase activity. Each sample was added with 100 μL of firefly luciferase working solution in order to detect the activity of firefly luciferase. Another 100 μL of renilla luciferase working solution was also added in order to detect the renilla luciferase activity. The firefly luciferase activity rate to renilla luciferase activity rate was used as the relative luciferase activity.

### RNA immunoprecipitation (RIP)

The binding of LINC00485 and argonaute 2 (AGO2) protein was verified and conducted according to the instructions of the RIP kit (Millipore Inc., Bedford, MA, USA). Part of the cell extract was used as the input, and the other part was incubated with the antibodies and magnetic beads. The magnetic beads-antibody complexes were re-suspended in 900 μL of RIP wash buffer. In order to collect the magnetic bead-protein complexes, the samples were put on to a magnetic base. RNAs in the samples and input were separately extracted after the detachment by protease K for the subsequent PCR detection. The antibodies for RIP included AGO2 (ab32381, 1:50, Abcam Inc., Cambridge, MA, USA), and immunoglobulin G (IgG) (1:100, ab109489, Abcam Inc., Cambridge, MA, USA) was used as the NC.

### RNA pull down

By following the instructions of the Magnetic RNA–Protein Pull-Down Kit (Pierce, Rockford, IL, USA), the biotinylated RNA was mixed with the structural Buffer at 1:500. After that, it was bathed in water at 95 °C for 2 min and on the ice for 3 min, with the magnetic beads being fully re-suspended after. The magnetic bead-RNA complex was washed with 500 μL of washing buffer 3 times and 10 μL of cell lysate was added. By adding 500 μL of RNA pull down washing buffer, the incubated magnetic bead-RNA–protein complex was washed. 10 μL of cell lysate was added in order to serve as the protein input. Following the measurement of the protein concentration, western blot analysis was performed in order to determine the protein expression.

### RNA isolation and quantitation

Total RNA was extracted with the miRNeasy Mini Kit (217004, QIAGEN, Duesseldorf, Germany). The primers of LINC00485, miR-195 and CHEK1 were designed and synthesized by Takara (Tokyo, Japan) (Table 1). After the primers were designed and synthesized, the RNA was reversely transcribed into complementary DNA (cDNA) in accordance to the instructions found in the PrimeScript RT kit (RR036A, Takara, Tokyo, Japan. By following the instructions in the SYBR^®^ Premix Ex TaqTM II kit (RR820A, TaKaRa, Tokyo, Japan), the fluorescence quantitative PCR was able to be carried out by using the ABI 7500 quantitative PCR appliance (7500, ABI, Thermo Fisher Scientific, Waltham, MA, USA). With glyceraldehyde-3-phosphate dehydrogenase (GAPDH) and U6 being used as the internal reference, the mRNA expression of target genes (LINC00485, miR-195, CHEK1) were calculated by using the relative quantitative method (2^−ΔΔCT^ method):

$${\Delta \Delta \text{Ct} = \Delta \text{Ct}}_{{\text{(experiment group)}}} - {\Delta \text{Ct}}_{{\text{(control group)}}} \text{,}\;{\Delta \text{Ct} = \text{Ct}}_{{\text{(target}\;\text{gene)}}} - \text{Ct}_{{\text{(internal reference)}}} \text{,}\;\text{mRNA expression of target gene = 2}^{{ - {\Delta \Delta \text{Ct}}}}.$$

**Table 1:** Primer sequences for RT-qPCR

Gene	Sequence (5′–3′)
LINC00485	F: CTCCAAGCAGGGGCTACAAA
	R: CCAGGAGCTCAGAAAGCCAA
miR-195	F: CGTAGCAGCACAGAAATATTGGC
	R: CCAGTCTCAGGGTCCGAGGTATTC
CHEK1	F: GACTGGGACTTGGTGCAAAC
	R: TGCCATGAGTTGATGGAAGA
GAPDH	F: CCCACTCCTCCACCTTTGAC
	R: ATGAGGTCCACCACCCTGTT
U6	F: ATTGGAACGATACAGAGAAGATT
	R: GGAACGCTTCACGAATTTG

*RT-qPCR* reverse transcription quantitative polymerase chain reaction, *LINC00485* long intervening noncoding 00485, *miR-195* microRNA-195, *CHEK1* checkpoint kinase 1, *GAPDH* glyceraldehyde-3-phosphate dehydrogenase, *F* forward, *R* reverse

### Western blot analysis

The total protein of cells was extracted using the radioimmunoprecipitation assay (RIPA) lysis buffer (R0010, Beijing Solarbio Science & Technology Co., Ltd., Beijing, China) containing phenylmethylsulfonyl (PMSF). Following the extraction period, 50 μg of protein was dissolved in 2× sodium dodecyl sulfate (SDS) loading buffer, and boiled 100 °C for 5 min. The protein was subjected to SDS–polyacrylamide gel electrophoresis and then transferred to a polyvinylidene fluoride (PVDF) membrane. This membrane was blocked by 5% skimmed milk for 1 h and incubated with diluted mouse anti-human primary antibodies CHEK1 (ratio of 1:10,000, ab40866), Bcl-2-associated X protein (Bax) (ratio of 1:5000, ab32503), B-cell CLL/lymphoma 2 (Bcl-2) (ratio of 1:1000, ab32124), HIF-1α (ratio of 1:2000, ab113642), and vascular endothelial growth factor (VEGF) (ratio of 1:5000, ab32152) overnight at a constant temperature of 4 °C. All the antibodies above were purchased from Abcam Inc. (Cambridge, MA, UK). The membrane was then incubated for 1 h with the secondary antibody horseradish peroxidase (HRP) labeled goat anti-mouse IgG (1:100, HA1003, Shanghai Yan Hui Biotechnology, Inc., Shanghai, China). After the 1 h incubation period, the membrane was then treated with the enhanced chemiluminescence (ECL) solution (ECL808-25, Biomiga, San Diego, CA, USA) for 1 min. The solution was abandoned, and the membrane was covered with plastic wrap and developed by using X-rays (36209ES01, Qcbio Science and Technologies Co., Ltd, Shanghai, China). GAPDH was used as the internal reference, and the ratio of the gray value of target band to the internal reference band was relative to the amount of protein.

### Cell counting kit-8 (CCK-8) assay

The cells were inoculated into a 96-well plate 48 h after transfection at a density of 2 × 10^3^ cells/well. Following a 16 h period, the culture medium was replaced by a fresh culture medium containing different concentrations of cisplatin (0, 0.5, 1, 2, 3 μg/mL), with five replicated wells being set at each concentration. Following a 48-h period of drug treatment, the culture medium was replaced by 100 μL of fresh culture medium containing 10% CCK-8, and underwent cultural incubation for 1 h. By using a multi-functional micro plate reader, The absorbance (A) was able to be examined at a 450 nm wavelength, with the inhibitory rate (IR) of cisplatin, which was held at different levels of concentration, being calculated using the following formula: IR (%) = (1 − Ai/A_0_) × 100% (Ai was the average absorbance of cisplatin groups at each concentration, and A_0_ was the average absorbance in the group without cisplatin).

### Colony formation assay

Cells were seeded into a 96-well plate at a density of 2.0 × 10^3^ cells/well. After being seeded for a period of 16 h, the culture medium was replaced by a fresh culture medium containing different concentrations of cisplatin (0, 0.5, 1, 2, 3 μg/mL), with five replicated wells each being set at a different concentration. Following a 48-h period of drug treatment, the cells were detached by 0.25% trypsin and resuspended in Dulbecco’s modified Eagles medium (DMEM), which contained 10% FBS. The cells were seeded into a 6-well plate at a density of 1 × 10^3^ cells per well, and then underwent incubation at a level of 5% CO_2_ at a temperature of 37 °C. Following the incubation period, the culture medium was discarded and the cells were fixed by 4% Polyoxymethylene and stained with Giemsa staining solution for a period of 10 min. Afterwards, the cells were slightly washed and air-dried. The clone cluster was counted directly with the naked eyes, and the rate of colony formation was calculated.

### Flow cytometry

All cell groups were seeded into a 6-well plate at a density of 10 × 10^5^ cells/well. Following a 16-h period, the culture medium was replaced by a fresh culture medium containing different concentrations of cisplatin (0, 0.5, 1, 2, 3 μg/mL), with five identical wells each being set at a different concentration. Following 48 h of drug treatment, the cells were centrifuged and re-suspended in 200 μL of binding buffer solution. After that, 10 μL of Annexin V-FITC (ab14085, Abcam Inc, Cambridge, MA, USA) and 5 μL of propidium iodide (PI) were gently mixed and underwent a 15-min period of incubation, while avoiding light. Afterwards, cells were added with 300 μL of binding buffer solution, and the cell apoptosis was detected via FCM at an excitation wavelength of 488 nm.

### Statistical analysis

Data were analyzed using SPSS 21.0 software (IBM Corp. Armonk, NY, USA). The measurement data were presented as mean ± standard deviation. Differences between two groups were analyzed by independent *t* test, and differences among multiple groups were analyzed by one-way analysis of variance (ANOVA). The enumeration data were represented by percentage, and analyzed by Chi square test. *p *< 0.05 was statistically significant.

## Results

### CHEK1 is a target gene of miR-195 and LINC00485 can specifically bind to miR-195

**Fig. 1:**
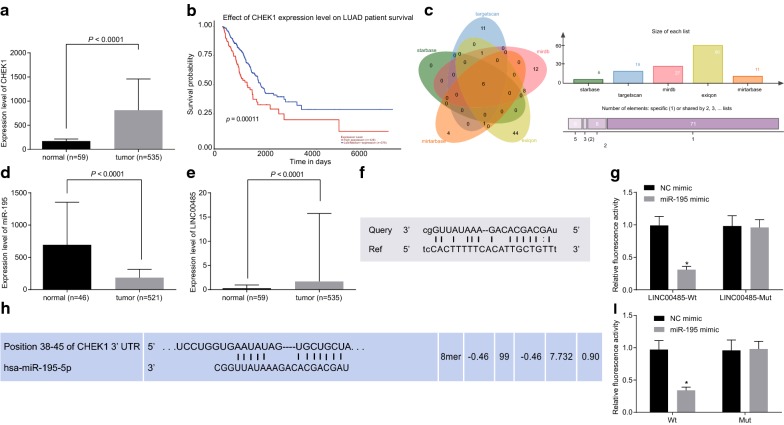
LINC00485 specifically binds to miR-195, and miR-195 targets CHEK1. **a** The expression of CHEK1 in LAC and normal control in TCGA database; **b** the survival of CHEK1 in LAC; **c** the miRNAs that targeted CHEK1 predicted by 5 biological online sites; **d** the expression of miRNAs in LAC and normal tissues in TCGA database; **e** the expression of LINC00485 in LAC and normal tissues in TCGA database; **f** the binding sites of LINC00485 and miR-195; **g** the luciferase activity of LINC00485-Wt and LINC00485-Mut without or with transfection of miR-195; **h** the binding sites of miR-195 and CHEK1; **i** luciferase activity of CHEK1-Wt and CHEK1-Mut without or with transfection of miR-195; *p < 0.05 vs. the NC. The enumeration data between two groups were analyzed by *t* test. All the experiments were detected 3 times independently, and the results were expressed as mean ± standard deviation. LINC00485, long intervening noncoding 00485; CHEK1, checkpoint kinase 1; Wt, wide type; Mut, mutant type; LAC, lung adenocarcinoma; TCGA, the Cancer Genome Atlas

The results of microarray analysis in TCGA database suggested that CHEK1 was highly expressed in LAC (Fig. 1a) and might be associated with LAC prognosis (Fig. 1b). By utilizing the Starbase database (http://starbase.sysu.edu.cn/index.php), Targetscan database (http://www.targetscan.org/vert_71/), Mirtarbase database (http://mirtarase.mbc.nctu.tw/php/search.php), Mirdb database (http://www.mirdb.org/) and Exiqon database (https://www.exiqon.com/ls/Pages/TPTSequenceInput.aspx?SessionPrefixKey=0a17e038-44fc-4f93-93-84aa-c326a29a073a), we found that miR-16-5p, miR-15a-5p, miR-195-5p, miR-497-5p, miR-15b-5p and miR-424-5p might target CHEK1 (Fig. 1c). By using the TCGA database and retrieving the expression data of miRNAs, it was found that only miR-195-5p was differentially expressed in LAC, with its own expression being lower when compared to that of the normal control (Fig. 1d). According to mircode web site (http://mircode.org/) and TCGA, the expression of LINC00485 was found to be visibly higher in LAC, when compared to that of the normal control (Fig. 1e). By utilizing the dual luciferase reporter gene assay, the target relationship of LINC00485 and miR-195, and that of miR-195 and CHEK1, were able to be verified. Co-transfection of miR-195/LINC00485-Wt was found to be able to lead to the reduction of the fluoresce signal (*p* < 0.05), while the results of Mut 3′ UTR transfection was similar to those without transfection (*p* > 0.05), thereby suggesting that LINC00485 specifically bound to miR-195 (Fig. 1f, g). When compared to that of the NC, the luciferase activity was reduced by miR-195/CHEK1-Wt co-transfection (*p* < 0.05), while also displaying no clear difference of the luciferase activity after being co-transfected with miR-195/CHEK1-Mut plasmid (*p* > 0.05). Therefore, miR-195 can target and bind to CHEK1, and CHEK1 was a target gene of miR-195 in turn (Fig. 1h, i).

### High expression of LINC00485 in A549 and H1299 cell lines

**Fig. 2:**
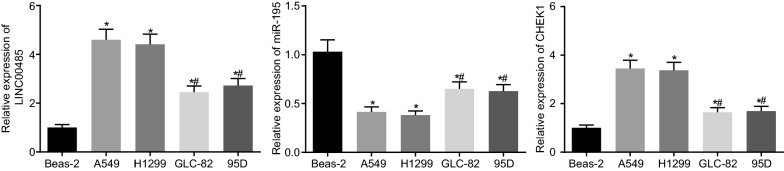
LINC00485 and CHEK1 are highly expressed, and miR-195 is poorly expressed in A549 and H1299 cells. *p < 0.05 vs. Beas-2B; ^#^*p* < 0.05 vs. A549 cells; the enumeration data between two groups were analyzed by Chi square test. All the experiments were detected 3 times independently, and the results were expressed as mean ± standard deviation. RT-qPCR, reverse transcription quantitative polymerase chain reaction

In order to screen out a LAC cell line that contained the highest expression of LINC00485, RT-qPCR revealed that (Fig. 2) when compared to that of the normal human epithelial cells Beas-2B, the expression of LINC00485 and CHEK1 was significantly higher, and that of miR-195 was lower in 4 LAC alpha cell lines (*p* < 0.05). Among the 4 human LAC cell lines, the LINC00485 and CHEK1 expression in A549 and H1299 cells was the highest, and that of miR-195 was the lowest (*p* < 0.05). Therefore, A549 and H1299 cells were selected for the subsequent experiments.

### LINC00485 competitively binds to miR-195 thereby up-regulating CHEK1

**Fig. 3:**
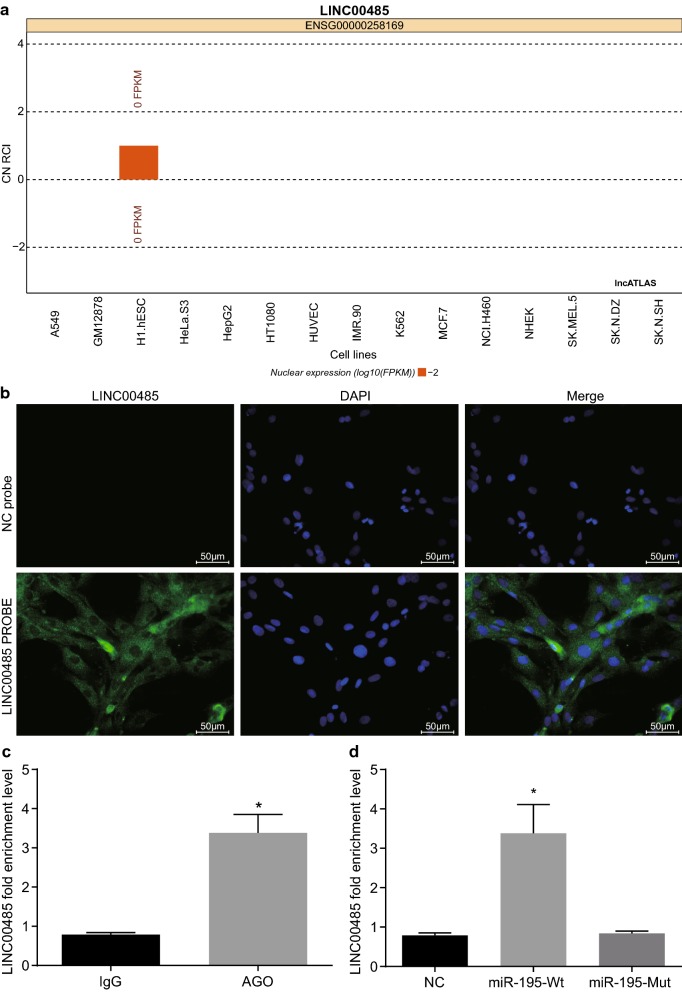
LINC00485 binds to miR-195. **a** The localization of LINC01234 predicted by the bioinformatics website; **b** the subcellular localization of LINC00485 in cells detected by FISH; **c** the relationship of LINC00467 and AGO2 detected by RIP (*p < 0.05 vs. IgG); **d** the relationship of LINC01082 and miR-195 detected by RNA pull down; *p < 0.05 vs. the NC group and the miR-195-Mut group. The enumeration data between two groups were analyzed by *t* test. All the experiments were detected 3 times independently, and the results were expressed as mean ± standard deviation. FISH, fluorescence in situ hybridization; RIP, RNA immunoprecipitation; AGO2, argonaute 2; IgG, immunoglobulin G; DAPI, 4′,6-diamidino-2-phenylindole

With an aim to test the relationship between LINC00485 and miR-195, FISH and RIP/RNA pull down were carried out. Bioinformatics website prediction results revealed that LINC00485 was mainly distributed in cytoplasm (Fig. 3a). The results from FISH revealed that LINC00485 was located in cytoplasm (Fig. 3b). Moreover, LINC00485 bound to AGO2, and LINC00485 bound to miR-195 (Fig. 3c). It suggested that LINC00485 could competitively bind to miR-195.

### Interference of LINC00485 or overexpression of miR-195 down-regulates CHEK1, Bcl-2, VEGF and HIF-1α expression, but up-regulates that of Bax in LAC cells

**Fig. 4:**
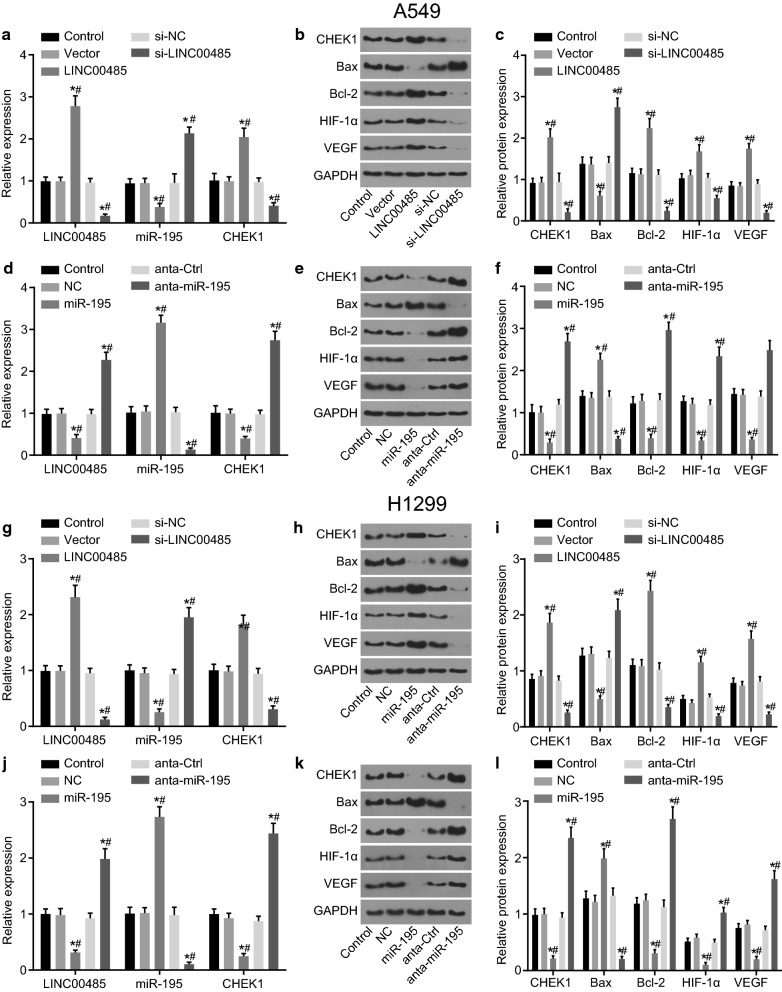
LINC00485 binds to miR-195 thereby up-regulating CHEK1, Bcl-2, VEGF and HIF-1α expression, and down-regulating Bax expression in LAC cells. **a** miR-195 and LINC00485 expression and the mRNA expression of CHEK1 in A549 cells after treatment of different LINC00485 expression examined by RT-qPCR, GAPDH and U6 as the internal references; **b**, **c** the protein bands and expression of CHEK1, Bax, Bcl-2, VEGF and HIF-1α in A549 cells with LINC00485 intervention examined by western blot analysis; **d** miR-195 and LINC00485 expression and the mRNA expression of CHEK1 in A549 cells after treatment of different miR-195 expression examined by RT-qPCR, GAPDH and U6 as the internal references; **e**, **f** the protein bands and expression of CHEK1, Bax, Bcl-2, VEGF and HIF-1α in A549 cells with miR-195 intervention examined by western blot analysis; **g** miR-195 expression and the mRNA expression of LINC00485 and CHEK1 in H1299 cells after treatment of different LINC00485 expression examined by RT-qPCR, GAPDH and U6 as the internal references; **h**, **i** the protein bands and expression of CHEK1, Bax, Bcl-2, VEGF and HIF-1α in H1299 cells with LINC00485 intervention examined by western blot analysis; **j** miR-195 and LINC00485 expression and the mRNA expression of CHEK1 in H1299 cells after treatment of different miR-195 expression examined by RT-qPCR, GAPDH and U6 as the internal references; **k**, **l** the protein bands and expression of CHEK1, Bax, Bcl-2, VEGF and HIF-1α in H1299 cells with miR-195 intervention examined by western blot analysis; *p < 0.05 vs. the control group; ^#^*p* < 0.05 vs. the vector, the si-NC groups or the NC, anta-Ctrl groups. Differences among multiple groups were analyzed by one-way ANOVA. All the experiments were detected 3 times independently, and the results were expressed as mean ± standard deviation. CHEK1, checkpoint kinase 1; Bax, Bcl-2-associated X protein; Bcl-2, B-cell CLL/lymphoma 2; VEGF, vascular endothelial growth factor; GAPDH, glyceraldehyde-3-phosphate dehydrogenase

In order to examine role of LINC00485 and miR-195 in LAC cells, A549 and H1299 cells were transfected with LINC00485, si-LINC00485, miR-195, or anta-miR-195. The results presented in Fig. 4a–c showed that the expression of LINC00485, CHEK1, Bcl-2, VEGF and HIF-1α in A549 cells was elevated, and the expression of miR-195 and Bax was reduced obviously in cells after transfection with LINC00485 (*p* < 0.05). In cells transfected with si-LINC00485, the expression of LINC00485, CHEK1, Bcl-2, VEGF and HIF-1α was down-regulated, while the expression of miR-195 and Bax was obviously up-regulated (*p* < 0.05). And the blank vector and si-NC did not induce any changes of the expression of LINC00485, miR-195, CHEK1, Bcl-2, VEGF, HIF-1α, and Bax (*p* > 0.05).

The effect of miR-195 expression on expression of related proteins in A549 cells was detected using RT-qPCR and western blot analysis (Fig. 4d–f). miR-195 down-regulated the expression of LINC00485, CHEK1 Bcl-2, VEGF, and HIF-1α, and up-regulated that of Bax (*p* < 0.05), while anta-miR-195 exhibited the opposite trend. The results of anta-Ctrl were similar to that of NC (*p* > 0.05). From the results above, it could be concluded that the silenced LINC00485 or overexpressed miR-195 could decrease the expression of CHEK1, Bcl-2, VEGF and HIF-1α while increase that of Bax.

Additionally, we repeated all the above-mentioned experiments in H1299 cell lines. The findings suggested that all the results obtained from H1299 cell lines were consistent with that from A549 cell lines (Fig. 4g–l).

### Enhanced miR-195 or inhibited LINC00485 promotes the inhibitory role of cisplatin on LAC cell proliferation

**Fig. 5:**
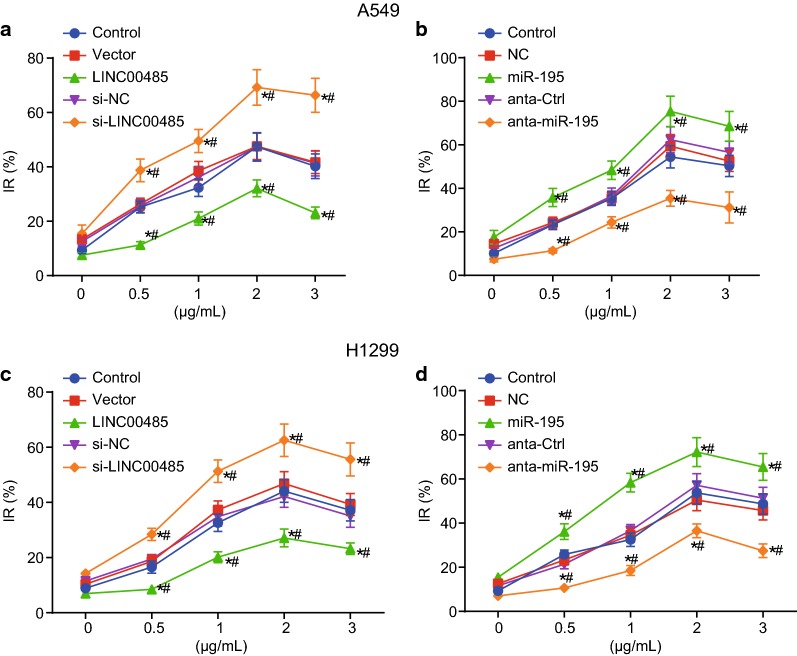
Overexpressed miR-195 or silenced LINC00485 promotes the inhibition of cisplatin on LAC cell proliferation. **a** Inhibitory rate of A549 cell growth with intervention of LINC00485; **b** inhibitory rate of A549 cell growth with intervention of miR-195; **c** inhibitory rate of H1299 cell growth with intervention of LINC00485; **d** inhibitory rate of H1299 cell growth with intervention of miR-195; *p < 0.05 vs. the control; ^#^*p* < 0.05 vs. cells transfected with blank vector, si-NC or the NC and anta-Ctrl. Differences among multiple groups were analyzed by one-way ANOVA. All the experiments were detected 3 times independently, and the results were expressed as mean ± standard deviation

The next step in the experiment was to investigate the effect that LINC00485 and miR-195 had on the inhibitory role of cisplatin in the proliferation of A549 and H1299 cells. While holding the same concentration levels of cisplatin, the inhibitory rate of cell growth in cells transfected with LINC00485 was lower, when compared to that of the control group (*p* < 0.05). Furthermore, in cells that were transfected with si-LINC0485 (p < 0.05), the inhibitory cell growth rate was visibly higher. The blank vector and the si-NC induced no changes of inhibitory rate (*p* > 0.05, Fig. 5a).

Within groups that had different levels of miR-195, the results of the CCK-8 assay are shown in Fig. 5b. In cells that had the same concentration of cisplatin, the inhibitory rate of A549 cell growth was higher in cells transfected with miR-195 (*p* < 0.05), while anta-miR-195 exhibited the opposite trend (*p* < 0.05). The results of anta-Ctrl were similar to that of NC (*p* > 0.05).

Moreover, all the above-mentioned experiments in H1299 cell lines were repeated. The findings suggested that all the results obtained from H1299 cell lines were consistent with that from A549 cell lines (Fig. 5c, d).

The results showed that different concentrations of cisplatin had certain inhibitory effects on cell proliferation, and that with higher levels of concentration, the effects became that much stronger. After reaching the optimal concentration, the inhibition of cisplatin on cell proliferation was gradually decreased. By restoring the original levels of miR-195 and suppressing LINC00485, the inhibition of cisplatin was promoted.

### Elevated miR-195 or suppressed LINC00485 promotes the inhibition of cisplatin on LAC cell colony formation

**Fig. 6:**
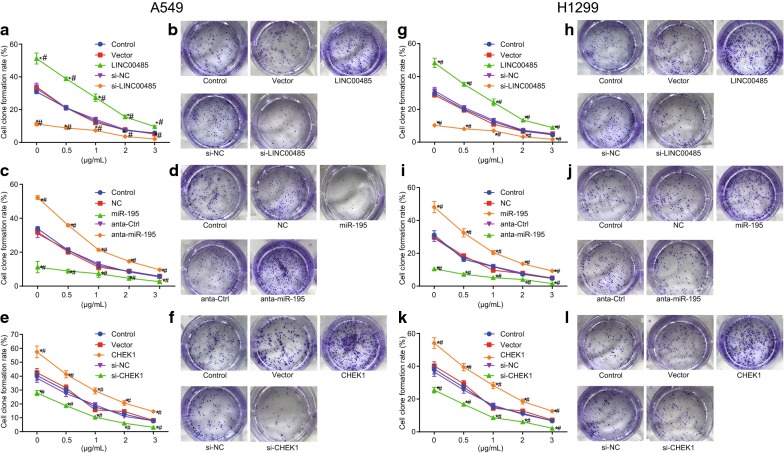
Restored miR-195 or LINC00485 silencing promotes the inhibitory function of cisplatin on colony formation in LAC cells. **a** The colony formation rate of A549 cells with intervention of LINC00485; **b** the colony formation of A549 cells treated by 3 μg/mL cisplatin with intervention of LINC00485; **c** the colony formation rate of A549 cells with intervention of miR-195; **d** the colony formation of A549 cells treated by 3 μg/mL cisplatin with intervention of miR-195; **e** the colony formation rate of A549 cells with intervention of CHEK1; **f** the colony formation of A549 cells treated by 3 μg/mL cisplatin with intervention of CHEK1; **g** the colony formation rate of H1299 cells with intervention of LINC00485; **h** the colony formation of H1299 cells treated by 3 μg/mL cisplatin with intervention of LINC00485; **i** the colony formation rate of H1299 cells with intervention of miR-195; **j** the colony formation of H1299 cells treated by 3 μg/mL cisplatin with intervention of miR-195; **k** the colony formation rate of H1299 cells with intervention of CHEK1; **l** the colony formation of H1299 cells treated by 3 μg/mL cisplatin with intervention of CHEK1; *p < 0.05 vs. the control; ^#^*p* < 0.05 vs. cells transfected with blank vector, si-NC, NC and anta-Ctrl. Differences among multiple groups were analyzed by one-way ANOVA. All the experiments were repeated 3 times independently, and the results were expressed as mean ± standard deviation

In order to explore the effect of LINC00485 and miR-195 on colony formation of A549 and H1299 cells, colony formation assay was performed. The higher concentration of cisplatin reflected lower colony formation rate. With the same concentration of cisplatin, the colony formation rate in cells transfected with LINC00485 was higher than that in control (*p* < 0.05) while the si-LINC00485 exhibited the opposite results (*p* < 0.05). The blank vector and si-NC induced no change of colony formation rate (*p* > 0.05, Fig. 6a, b).

The results of different miR-195 expression on the colony formation of A549 cells are showed in Fig. 6c, d. With the same concentration of cisplatin, the colony formation rate in cells transfected with miR-195 was lower than that in the control group (*p* < 0.05) while anta-miR-195 showed increased colony formation rate (*p* < 0.05). The results of anta-Ctrl were consistent with NC (*p* > 0.05). The results showed that up-regulated miR-195 or si-LINC00485 suppressed the cell colony formation, and different concentrations of cisplatin had inhibitory roles on the colony formation. The higher the concentration was, the stronger the inhibitory effect was.

The results of different CHEK1 expression on the colony formation of A549 cells are showed in Fig. 6e, f. The higher the concentration of cisplatin reflected lower colony formation rate. With the same concentration of cisplatin, the colony formation rate in cells transfected with CHEK1 was higher than that in the control group (*p* < 0.05) while si-CHEK1 showed significantly decreased colony formation rate (*p* < 0.05). The blank vector and si-NC showed no difference of colony formation rate (*p* > 0.05).

Furthermore, we repeated all the above-mentioned experiments in H1299 cell lines. The findings suggested that all the results obtained from H1299 cell lines were consistent with that from A549 cell lines (Fig. 6g–l).

Therefore, the overexpression of miR-195 and si-LINC00485 has the ability to inhibit the function of cisplatin on colony formation.

### miR-195 and silenced LINC00485 enhances the stimulation of cisplatin on LAC apoptosis

**Fig. 7:**
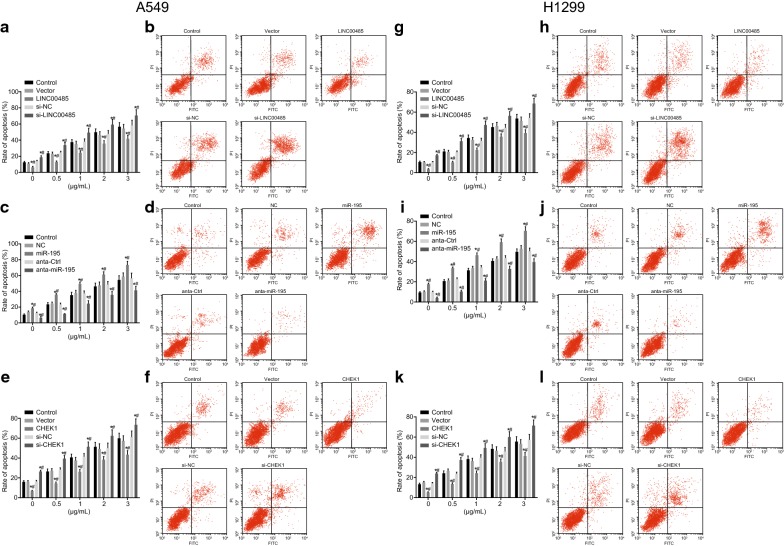
Up-regulated miR-195 or down-regulated LINC00485 promotes the apoptosis of LAC cells treated with cisplatin. **a** The apoptosis rate of A549 cells with intervention of LINC00485; **b** the apoptosis of A549 cells treated by 3 μg/mL cisplatin with intervention of LINC00485; **c** the apoptosis rate of A549 cells with miR-195 intervention; **d** the apoptosis of A549 cells treated by 3 μg/mL cisplatin with intervention of miR-195; **e** the apoptosis rate of A549 cells with intervention of CHEK1; **f** the apoptosis of A549 cells treated by 3 μg/mL cisplatin with intervention of CHEK1; **g** the apoptosis rate of H1299 cells with intervention of LINC00485; **h** the apoptosis of H1299 cells treated by 3 μg/mL cisplatin with intervention of LINC00485; **i** the apoptosis rate of H1299 cells with intervention of miR-195; **j** the apoptosis of H1299 cells treated by 3 μg/mL cisplatin with intervention of miR-195; **k** the apoptosis rate of H1299 cells with intervention of CHEK1; **l** the apoptosis of H1299 cells treated by 3 μg/mL cisplatin with intervention of CHEK1; *p < 0.05 vs. the control; ^#^*p* < 0.05 vs. cells transfected with blank vector, si-NC, NC and anta-Ctrl. Differences among multiple groups were analyzed by one-way ANOVA. All the experiments were detected 3 times independently, and the results were expressed as mean ± standard deviation

To examine the role of LINC00485 and miR-195 in cisplatin’s regulatory function on the apoptosis of A549 and H1299 cells, Annexin V-FITC/PI double staining was conducted in the cells transfected with different LINC00485 expressions followed by the treatment with 3 μg/mL cisplatin. The higher the concentration of cisplatin reflected higher apoptosis rate. With the same concentration of cisplatin, the apoptosis of A549 cells transfected with LINC00485 was slower while that in cells transfected with si-LINC00485 was higher (*p* < 0.05). The blank vector and the si-NC exhibited no change in cell apoptosis (*p* > 0.05, Fig. 7a, b).

Meanwhile, the apoptosis rate of A549 cells transfected with different expressions of miR-195 and 3 μg/mL cisplatin were measured (Fig. 7c, d). With the same concentration of cisplatin, cell apoptosis in cells transfected with miR-195 was enhanced while in the anta-miR-195 group the rate was reduced (*p* < 0.05). The result of anta-Ctrl was similar to that in the NC group (*p* > 0.05).

Furthermore, the apoptosis rate of A549 cells transfected with different expressions of CHEK1 and 3 μg/mL cisplatin were measured (Fig. 7e, f). The higher the concentration of cisplatin reflected higher apoptosis rate. With the same concentration of cisplatin, A549 cell apoptosis transfected with CHEK1 was reduced while in the si-CHEK1 group, the apoptosis was increased (*p* < 0.05). The blank vector and si-NC showed no difference in cell apoptosis (*p* > 0.05).

Furthermore, we repeated all the above-mentioned experiments in H1299 cell lines. The findings suggested that all the results obtained from H1299 cell lines were consistent with that from A549 cell lines (Fig. 7g–l).

The results showed that the up-regulation of miR-195 or the silence of LINC00485 could promote cell apoptosis. Different concentrations of cisplatin had a certain promotional effect on cell apoptosis, and the higher concentration reflected stronger effect.

### CHEK1 reverses effects of miR-195 on the LAC cells

**Fig. 8:**
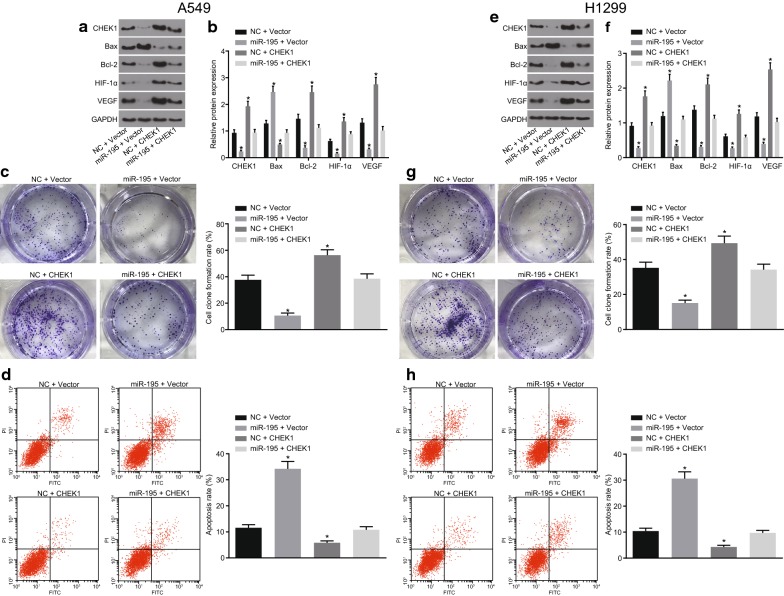
CHEK1 reverses the impacts of miR-195 on LAC cells. **a**, **b** The protein bands and expression of CHEK1, Bcl-2, VEGF, HIF-1α and Bax in A549 cells; **c** the colony formation of A549 cells; **d** the apoptosis of A549 cells; **e**, **f** the protein bands and expression of CHEK1, Bcl-2, VEGF, HIF-1α and Bax in H1299 cells; **g** the colony formation of H1299 cells; **h** the apoptosis of H1299 cells; *p < 0.05 vs. cells co-transfected with NC + vector. Differences among multiple groups were analyzed by one-way ANOVA. All the experiments were detected 3 times independently, and the results were expressed as mean ± standard deviation

In order to investigate the anaplerotic reaction of CHEK1 to miR-195 in A549 and H1299 cells, miR-195 and CHEK1 were co-transfected, respectively. When only transfected with miR-195, the protein expression of Bax in A549 cells was up-regulated, while that of CHEK1, Bcl-2, VEGF and HIF-1α were found to be down-regulated (*p* < 0.05). Nonetheless, when only CHEK1 was transfected, the expression of the proteins tended to be the opposite. Moreover, following the transfection period of miR-195 and CHEK1, the protein expression was similar, when compared to that of the NC group (Fig. 8a, b). As depicted in Fig. 8c, d, co-transfection with miR-195 and CHEK1 reversed the promoting effects on the apoptosis rate and the inhibitory effects on the colony formation rate of A549 cells by only transfection with miR-195 (*p* < 0.05).

Furthermore, all the above-mentioned experiments in H1299 cell lines were repeated multiple times. These findings suggested that all the results obtained from H1299 cell lines were consistent with that from A549 cell lines (Fig. 8e–h).

### miR-195 reverses effects of LINC00485 on the LAC cells

**Fig. 9:**
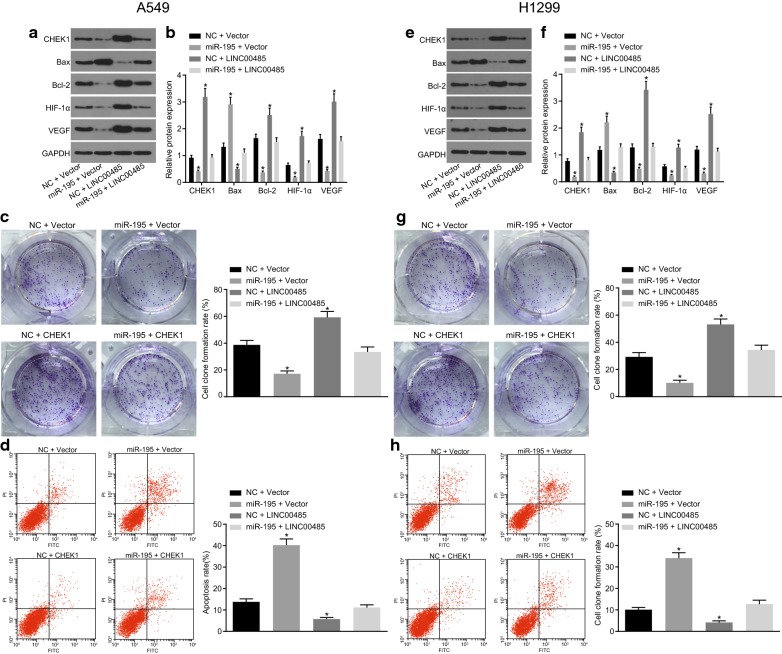
miR-195 reverses effects of LINC00485 on the LAC cells. **a**, **b** The protein bands and expression of CHEK1, Bcl-2, VEGF, HIF-1α and Bax in A549 cells; **c** the colony formation of A549 cells; **d** the apoptosis of A549 cells; **e**, **f** the protein bands and expression of CHEK1, Bcl-2, VEGF, HIF-1α and Bax in H1299 cells; **g** the colony formation of H1299 cells; **h** the apoptosis of H1299 cells; *p < 0.05 vs. cells co-transfected with NC + vector. All the experiments were detected 3 times independently, and the results were expressed as mean ± standard deviation. Differences among multiple groups were analyzed by one-way ANOVA

When only transfected with LINC00485, the protein expression of Bax in A549 cells was down-regulated, while that of CHEK1, Bcl-2, VEGF and HIF-1α were found to be up-regulated (*p* < 0.05). Nonetheless, when only miR-195 was transfected, the expression of the proteins tended to be the opposite (*p* < 0.05). Moreover, following the transfection period of LINC00485 and miR-195, the protein expression was similar, when compared to that of the NC (Fig. 9a, b, e, f).

The apoptotic rate and colony formation after co-transfected LINC00485 and miR-195 in LAC cells were further detected (Fig. 9c, d, g, h). The results showed that when only transfected with LINC00485, the clone formation rate increased, and the cell apoptosis rate decreased, while it was reciprocal after miR-195 treatment (*p* < 0.05). After LINC00485 and miR-195 co-treatment, the effects were neutralized, which showed that the effect of LINC00485 on cell functions was reversed by miR-195.

## Discussion

Lung cancer represents a common cancer worldwide, and LAC is the most often histological type with the highest degree of diversity [11]. Our study explored roles of LINC00485 as competitive endogenous RNA (ceRNA) of miR-195 in the chemotherapy sensitivity of LAC cells by regulating CHEK1. Consequently, we found that silencing of LINC00485 and restored miR-195 promoted the inhibitory roles of cisplatin on LAC cell proliferation, clone information and enhanced the apoptosis via decreased CHEK1.

To begin with, CHEK1 was found to be highly expressed, while miR-195 was poorly expressed in LAC. We also found that miR-195 targeted CHEK1. Consistent with our study, Liu et al. also demonstrate decreased miR-195 and increased CHEK1 in lung tumors and that CHEK1 is a target gene of miR-195, which down-regulates CHEK in lung cancers cells [9]. Similarly, the reduced miR-195 expression has been demonstrated in NSCLC, and miR-195 targets cyclin D3 and survivin to mediate tumorigenesis of NSCLC [17, 18]. In addition, miR-195 modulates response of NSCLC cells to microtubule-targeting agents (MTAs) through binding to CHEK1, and CHEK1 silencing synergized with MTAs suppressing the development of NSCLC [19]. Therefore, overexpression of miR-195 and inhibited CHEK1 exerted their therapeutic potential in the LAC progression.

ceRNA is identified as an important class of post-transcriptional regulator altering gene expression by miRNAs [20]. Recently, it is found that lncRNAs can act as ceRNAs to mediate gene expression, involving the tumor progression [21]. In the subsequent experiments, it was reported that LINC00485 acted as a ceRNA of miR-195 and induced the cisplatin resistance in LAC. Another study, Wu et al. also demonstrate that silenced lncRNA plasmacytoma variant translocation 1 (PVT1) strengthens the radio-sensitivity in NSCLC through down-regulating miR-195 as a ceRNA [22]. In addition, miR-137 and miR-27a also regulate cisplatin resistance in LAC [23, 24]. Moreover, silencing of lncRNA urothelial cancer-associated 1 is reported to enhance the cisplatin chemosensitivity in tongue squamous cell carcinoma cells [20].

Moreover, in this experiment, it was shown that the silencing of LINC00485 had the ability to elevate the expression of miR-195, and promote the inhibition that cisplatin had on cell proliferation, while at the same time, increasing the apoptosis rate in LAC cells. Another study, Ye et al, showed that the elevation of miR-195 levels played an important role in gastric cancer by promoting chemotherapy sensitivity of cisplatin, and by effectively upgrading the overall survival rate among patients with gastric cancer [15]. In the experimentation process, it was shown that miR-195 had a suppressive effect on cell proliferation, migration and invasion by binding to MYB and HDGF in NSCLC [25, 26]. Furthermore, the down-regulation of LINC00485 and the up-regulation of miR-195 could decrease the expression of CHEK1, and expression of apoptosis-related genes and angiogenesis-related genes Bcl-2, VEGF and HIF-1α, while also increasing the rate of apoptosis-related gene Bax in in LAC cells. In a previous study, it was shown that the inhibition of CHEK1 may reduce the progression rate of Lkb1-deficient LAC [27]. Furthermore, Xiang et al. demonstrated that 125I seed irradiation promotes the apoptosis rate and suppresses angiogenesis through the down-regulation of the expression of HIF-1α and VEGF in LAC cells [28]. Moreover, chronic exposure to exogenous matrilysin induces chemo-resistance and enhances Bcl-2 level in A549 LAC cells [29]. Overall, the silencing of LINC00485 or the restoration of miR-195 enhanced the cisplatin chemotherapy in LAC cells. By up-regulation of LINC00485 or down-regulation of miR-195, CHEK1 increased cell proliferation, anti-apoptosis, invasion and angiogenesis [30–32], while down-regulation of CHEK1 inhibited cell proliferation, anti-apoptosis, invasion and angiogenesis. Thus, CHEK1 plays an important role in LAC cell cycle, apoptosis and angiogenesis.

## Conclusions

Taken together, in the current study, we find that LINC00485 works as a ceRNA against miR-195 thereby up-regulating CHEK1 in LAC cells. By binding to miR-195, LINC00485 reduces chemotherapy sensitivity of cisplatin in LAC cells, thus promoting proliferation while inhibiting apoptosis of LAC cells. LINC00485 can be regarded as a new target for improving prognosis and the treatment of LAC. Our study provides a potential helpful method to treat LAC, but further in vitro experiments with more tumor samples or in vivo experiments should be conducted to expound the hypothesis in future.

## Data Availability

The datasets generated and/or analysed during the current study are available.

## Abbreviations

lncRNAs: long non-coding RNAs
miR-195: microRNA-195
LAC: lung adenocarcinoma
CHEK1: checkpoint kinase 1
NSCLC: non-small cell lung cancer
CT: computed tomography
miRNAs: microRNAs
TCGA: the Cancer Genome Atlas
FDR: false positive discovery
DEGs: differentially expressed genes
RPMI: Roswell Park Memorial Institute
NC: negative control
MEM: minimal essential medium
FISH: fluorescent in Situ Hybridization
PBST: phosphate-buffered saline/Tween
DAPI: diamidino-2-phenylindole
3′ UTR: 3′-untranslated region
Wt: wide-type
Mut: mutant-type
RIP: RNA immunoprecipitation
AGO2: argonaute 2
IgG: immunoglobulin G
Cdna: complementary DNA
GAPDH: glyceraldehyde-3-phosphate dehydrogenase
PMSF: phenylmethylsulfonyl
SDS: sodium dodecyl sulfate
PVDF: polyvinylidene fluoride
VEGF: vascular endothelial growth factor
HRP: horseradish peroxidase
ECL: electrochemiluminescence
CCK-8: cell counting kit-8
A: absorbance
IR: inhibitory rate
DMEM: Dulbecco’s modified Eagles medium
FBS: fetal bovine serum
PI: propidium iodide
ANOVA: one-way analysis of variance
ceRNA: competitive endogenous RNA
PVT1: plasmacytoma variant translocation 1
